# Colorectal Cancer in Ulcerative Colitis: Mechanisms, Surveillance and Chemoprevention

**DOI:** 10.3390/curroncol29090479

**Published:** 2022-08-25

**Authors:** Wenqian Li, Tiantian Zhao, Dacheng Wu, Jiajia Li, Mei Wang, Yunyun Sun, Sicong Hou

**Affiliations:** 1 Department of Gastroenterology, Affiliated Hospital of Yangzhou University, Yangzhou University, Yangzhou 225000, China; 2 Department of Clinical Medicine, Medical College, Yangzhou University, Yangzhou 225001, China

**Keywords:** ulcerative colitis-associated colorectal cancer, risk factors, cell signaling, surveillance, chromoendoscopy

## Abstract

Patients with ulcerative colitis (UC) are at a two- to three-fold increased risk of developing colorectal cancer (CRC) than the general population based on population-based data. UC-CRC has generated a series of clinical problems, which are reflected in its worse prognosis and higher mortality than sporadic CRC. Chronic inflammation is a significant contributor to the development of UC-CRC, so comprehending the relationship between the proinflammatory factors and epithelial cells together with downstream signaling pathways is the core to elucidate the mechanisms involved in developing of CRC. Clinical studies have shown the importance of early prevention, detection and management of CRC in patients with UC, and colonoscopic surveillance at regular intervals with multiple biopsies is considered the most effective way. The use of endoscopy with targeted biopsies of visible lesions has been supported in most populations. In contrast, random biopsies in patients with high-risk characteristics have been suggested during surveillance. Some of the agents used to treat UC are chemopreventive, the effects of which will be examined in cancers in UC in a population-based setting. In this review, we outline the current state of potential risk factors and chemopreventive recommendations in UC-CRC, with a specific focus on the proinflammatory mechanisms in promoting CRC and evidence for personalized surveillance.

## 1. Introduction

Ulcerative colitis (UC) is one form of inflammatory bowel disease (IBD) that commences in the rectum but often extends proximally to involve additional areas of the colon, it is characterized by a relapsing and remitting course [1]. Symptoms of UC depend on the extent and severity of the disease and include bloody diarrhea, rectal bleeding, tenesmus, urgency and fecal incontinence [2]. Patients with long-standing UC tend to develop serious complications, of which colorectal cancer (CRC) is well described [3,4,5]. UC patients developing CRC have a poor prognosis and a higher proportion of mucinous and signet ring cell carcinoma, making it vital to identify high-risk patients and conduct appropriate monitoring [6,7]. Similar to sporadic CRC, UC-CRC develops with a sequence of molecular events, including alterations of p53, adenomatous polyposis coli (APC) and K-ras; however, the sequence of events is different between sporadic CRC and UC-CRC. Predominantly, the loss function of APC is an early event in adenoma formation, but it occurs later in the pathogenesis of UC-CRC, whereas p53 is the opposite [8]. On the other, UC-CRC develops with an inflammatory–dysplasia–carcinoma sequence rather than the typical adenoma–carcinoma sequence. In addition, signaling pathways such as phosphatidylinositol-3-kinase (PI3K)/AKT and Wnt/β-catenin have been extensively studied in sporadic CRC [9], but these do not seem to have been given sufficient attention in UC-CRC. By contrast, activation of nuclear factor-κB (NF-κB), signal transducer and activator of transcription 3 (STAT3) induced by cytokines are prominent contributors to the UC-CRC development. Clinical studies have emphasized that the risk of developing CRC is higher in patients with specific risk factors. According to the stratification of risk factors, the concrete plan of cancer monitoring for UC patients is formulated, which can effectively distinguish low-risk patients from high-risk patients, so as to carry out effective individualized monitoring [10,11]. During the monitoring process, the new advanced endoscopic technologies are important diagnostic tools for the recognition of dysplasia and carcinoma, attaining higher efficiency and better optical characterization of histology [1,2,12]. However, because of the limitations of various techniques, the selection of suitable techniques and biopsy approaches remain to be discussed further. Finally, for clinicians, establishing a chemoprotective benefit with the use of medical therapy should be of paramount importance synchronously, the effects of that on CRC risk after treatment being reported in a number of clinical studies [13,14]. In this review, we outline from basic to clinical in regard to UC-CRC, providing a more up-to-date understanding of the risk prediction, pathogenesis, surveillance strategies and chemoprotection of UC-CRC.

## 2. Risk Factors of UC-CRC

Knowledge of risk factors for CRC is influential to identify UC patients who need frequent surveillance or intense treatment, namely anatomical extent of colonic involvement, duration of disease, concurrent primary sclerosing cholangitis (PSC) and family history of CRC. In addition, colonic strictures, postinflammatory polyps (PIPs), gender, age at diagnosis, severity of inflammation and dysplasia are also risk factors for CRC, delineating UC patients that should be enrolled in an intensive surveillance program [2,10,11,15].

### 2.1. Disease-Related Factors

Patients with UC are at higher risk of developing CRC, concerning disease extent and duration being two of the most prominent risk factors. As early as 1990, Ekbom et al. conducted a population-based cohort of 3117 patients with UC and discovered that the standardized incidence ratio (SIR) of CRC was 14.8 for patients with pancolitis (95% confidence interval (CI) 11.4–18.9), compared with 2.8 for left-sided colitis (95% CI 1.6–4.4) and 1.7 for proctitis (95% CI 0.8–3.2), suggesting pancolitis is a strong risk factor for CRC [16]. This conclusion laid the foundation for the anatomical extent of colitis as an independent risk factor and was confirmed by later research [17,18]. The longer duration of UC, another crucial risk factor for UC-CRC, was verified by a cohort study of 504 UC patients, in which stratified data manifested a cumulative incidence of CRC after a disease duration of 10 years was 1%; 20 years, 3%; 30 years, 7% [19], indicating that the longer the duration of UC is, the greater the risk of CRC [20,21]. One possible explanation is that long-term inflammatory stimulation can lead to the release of inflammatory cytokines, immune dysfunction and the alteration of epigenetic inheritance, thus leading to cancer, which again emphasizes the significant role of chronic inflammation in the pathogenesis of UC-CRC [22,23]. In a retrospective, longitudinal study of 1911 IBD patients, those with concomitant PSC were relevant to an estimated two-fold significantly higher independent risk of developing advanced colorectal neoplasia (aCRN) compared with those with IBD only [24]. Additionally, anatomic structural alterations to the colon, including strictures and PIPs, were reported to be connected with the increased risk of CRC in earlier studies [25,26]. In contrast, these connections have not been substantiated in recent years. The possible explanation for the difference is that patients with strictures and PIPs have more severe histologic inflammation, showing that they are related to the inflammatory burden, referred to as structural markers of cumulative inflammatory burden, they themselves are not independent risk factors for CRN [27,28,29]. Accordingly, their existence alone does not necessarily undergo heightened colonoscopy surveillance. Multicenter, prospective studies are needed to confirm these findings, particularly in patients with UC-associated colorectal strictures and PIPs.

### 2.2. Patient-Related Factors

In a nationwide population-based cohort study, the hazard ratio (HR) of UC in CRC incidence in females was 0.63 (95% CI 0.60–0.65), demonstrating that the risk of CRC in males was higher than that in females [30]. This gender difference resembles what has been observed for sporadic cases of CRC, which may be explained by the protective effect of estrogens on the development of CRC [31]. Although many analyses support family history of CRC as a well-established risk factor in healthy individuals, little is known about its role in the development of IBD. A cohort of 9505 individuals with IBD reported that a family history of CRC was linked to a nearly eight-fold increase in CRC risk (SIR 7.9, 95% CI 1.6–14.3), compared with the background population [32]. Consistently, in a retrospective cohort study, family history of CRC was noted as the risk factor for UC-CRC directly (HR 12.992, 95% CI 1.611–104.7, *p* = 0.02) [33]. The risk of CRC varied according to the age at the initial diagnosis of UC. A relative risk of patients diagnosed in childhood (0–19 years old) was 43.8, followed by those diagnosed in young (20–39 years old) was 2.65 [34], indicating that young age at onset of UC has been reported as an independent risk factor for CRC.

### 2.3. Pathology-Related Factors

Since UC-CRC develops through an inflammation–dysplasia–carcinoma pathway, dysplasia in UC is a vital risk factor in the development of CRC, both high-grade dysplasia (HGD) and low-grade dysplasia (LGD) [35,36]. This deciphers that most international societies promote dysplasia surveillance in long-standing UC [10,35,37]. Given the unquestionable role of intestinal inflammation in cancer progression, the severity of inflammation has exerted a profound impact on the occurrence of CRN among patients with UC [38,39]. Nevertheless, their design had a limitation, that is, using a single value of inflammation for each subject’s history cannot reflect changes in inflammation status throughout the disease. Therefore, the cumulative inflammatory burden (CIB), a new formula for describing persistent inflammation, was proposed in a longitudinal cohort study. CIB was defined as: each histological inflammatory activity (HIA) multiplied by the surveillance interval in years and it was significantly associated with CRN evolution (HR 1.07 per one-unit increase in CIB, 95% CI 1.04–1.11, *p* < 0.001) [40]. The conclusion that CIB was an index to predict the development of CRN in UC patients was validated by Olivia et al., which is consistent with the previous one [41]. The results of these trials will be bound to provide new evidence in the near future for an optimal control of inflammation in UC patients to prevent cancer occurrence.

## 3. Mechanisms of Colorectal Cancer in Ulcerative Colitis

**Figure 1:**
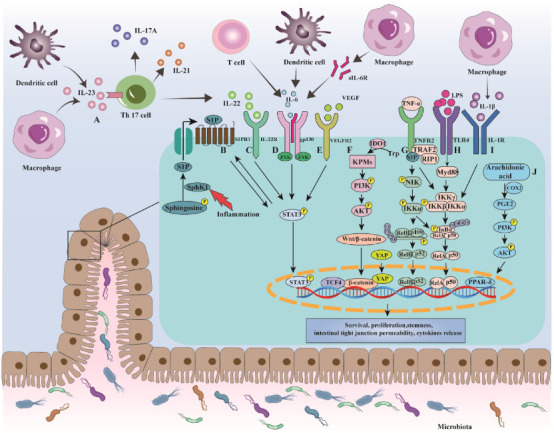
The involvement of cytokine-related signaling pathways in colitis-associated CRC. (**A**) Binding of IL-23 to IL-23R activates the downstream JAK/STAT pathway to release IL-17A, IL-21, IL-22 from Th17 cells, exerting proinflammatory and protumor effects. (**B**–**E**) Several factors such as S1P, IL-22, IL-6, VEGF contribute to phosphorylation of STAT3 that upregulates Bcl-x and Caspase 3, downregulate Bcl-xl to inhibit apoptosis. (**F**) Kynurenine pathway metabolites rapidly activate PI3K/AKT signaling which then promotes β-catenin translocate to the nucleus, where β-catenin can form transcription complex with YAP and TCF4. (**G**–**I**) Proinflammatory stimuli including TNF-α, IL-1β and LPS can participate in tumor initiation and development through boosting the downstream molecule NF-κB activity. (**J**) PGE2 activates PI3K/AKT/PPAR-δ signaling to promote cell survival, thus increasing tumor burden.

In the background of inflammation, cells of the innate immune system are activated and release various reactive oxygen and nitrogen species (RONS), including superoxide, hydroxyl radical, hydrogen peroxide, singlet oxygen, nitric oxide, nitrogen dioxide and peroxynitrite, among others [42]. As the basis of mutation, inflammation-induced RONS react with DNA to cause DNA damage (e.g., single- and double-strand breaks, nucleobase oxidation, nucleotide modification) [43]. For example, guanine is the most readily oxidized of the four DNA bases in UC-CRC; its oxidation can generate 8-oxo-7,8-dihydro-2′-deoxyguanosine (8oxoG), which leads to G:C-T:A transversions through DNA replication [44,45]. Moreover, radicals can hydrolyze the phosphodiester backbone, which makes a single strand break [44]. This DNA damage may change the coding and regulatory sequence of genes, such as p53, APC and DPC4 [46]. Hussain et al. explored the most common mutated gene p53 in the early stage of UC-associated dysplastic lesions; they observed that the CpG site of codon 248 and the third base at codon 247 had a higher frequency of G-to-A and C-to-T transition, respectively [47]. Subsequently, with the participation of gut microbiota, multiple pathways including NF-κB, IL-6/STAT3, interleukin-23 (IL-23)/T helper type 17 (Th17), cyclooxygenase-2 (COX-2)/Prostaglandin E2 (PGE2) and Wnt/β-catenin were activated for initiation and development of UC-CRC [48,49] (Figure 1).

### 3.1. NF-κB

NF-κB is considered one of the main regulatory components involved in UC-CRC initiation [49], consisting of five members named p65 (RelA), c-Rel, RelB, p50 and p52. In a state of rest, the motif within the amino acid sequence of NF-κB is masked by small inhibitory molecules called IκBα, IκBβ and IκBγ, inactivating NF-κB in the cytoplasm [43]. Normally, proinflammatory stimuli including tumor necrosis factor (TNF-α), IL-1β and bacterial cell wall components lead to the activation of IκB kinase (IKK) through canonical signaling, which could degrade IκBα protein, thereby exposing the nuclear localization signals (NLS) on RelA and inducing nuclear translocation of NF-κB (RelA-p50 heterodimers) [50].

Concretely, NF-κB signaling can be activated through TNF-α mediated by the NF-κB-inducing kinase (NIK)/IKK-α axis, which increases myosin light chain kinase (MLCK) expression, resulting in the breakdown of tight junctions (TJs) and increased tumorigenic cytokines [51,52] (Figure 1G). In addition, sphingosine-1-phosphate (S1P) is considered to be involved in the activation of NF-κB through TNF-α (Figure 1G). Mechanically, S1P specifically binds to the N-terminal RING domain of TNF receptor-associated factor 2 (TRAF2) and stimulates ubiquitin ligase activity of TRAF2, then dramatically increasing polyubiquitination of receptor interacting protein 1 (RIP1). This results in recruitment and phosphorylation of the IKK complex, leading to NF-κB dimer liberating from IκBα, producing IL-6 and severe inflammation [53,54]. Additionally, NF-κB was reported to be activated through IL-1β mediated by mitogen-activated protein kinase kinase-1 (MEKK-1)/IKKβ axis and also caused a progressive increase in MLCK expression, subsequently leading to an increased intestinal TJs permeability [55,56] (Figure 1I). The role of IL-1β was emphasized by Yin et al., who showed that FL-BsAb1/17, an antibody targeting IL-1β, limited the inflammatory cascade and reduced oxidative stress in mice with dextran sulfate sodium (DSS)-induced colitis [57]. Furthermore, the interaction between lipopolysaccharide (LPS) and toll-like receptor 4 (TLR4)/ myeloid differentiation factor 2 (MD2) complex could trigger NF-κB pathway, which may induce the expression of proinflammatory mediators and cytokines (e.g., nitric oxide synthase (NOS), TNF-α), thus finally perpetuating local tissue damage and promoting tumor growth [58] (Figure 1H). It is worth mentioning that NF-κB signaling can be prolonged by mutant p53, which causes persistent tissue damage and increases its probability of progressing toward a tumor [59].

Indeed, NF-κB in UC-CRC also contributes to tumorigenesis by promoting cell proliferation and angiogenesis. For instance, knockdown of C-X-C motif ligand 8 (CXCL8) or C-X-C chemokine receptor 1 (CXCR1) inhibits tumor growth and decreases vessel density in an animal model. CXCL8 and CXCR1 were thought to be regulated by NF-κB and their combination drove tumorigenesis by enhancing cell cycle control and angiogenesis [60].

### 3.2. IL-6/STAT3

IL-6 is a critical cytokine regulated by NF-κB because it exerts a synergistic proinflammatory and protumor function with NF-κB in UC-CRC. In detail, the binding of IL-6 to the soluble form of IL-6 receptor (sIL-6R) induces gp130 dimerization, which allows Janus kinase (JAK) activation that in turn phosphorylate several tyrosine residues in the cytoplasmic tail of gp130, providing docking sites for STAT3 [61] (Figure 1D). Phosphorylated STAT3 is translocated into the nucleus where it could inhibit apoptosis by decreasing expression of Bax and Caspase 3, increasing expression of Bcl-xl [62], thereby driving tumorigenesis [61]. Several animal experiments have validated this pathway. For example, IL-6-ablated mice and STAT3-deficient mice exposed to azoxymethane (AOM)/DSS exhibited reduced tumor numbers, size and proliferation [63,64]. Moreover, in colitis-associated premalignant cancer (CApC)-affected mice, the mRNA levels of gp130 and phosphorylation of STAT3 were augmented in the mucosal epithelial cells. Meanwhile, soluble gp130Fc (sgp130Fc)—a dimerized fusion protein of sgp130 that explicitly suppresses the activation of gp130 via the IL-6/sIL-6R complex—was demonstrated to lower the incidence and number of tumors [65]. Similar data were shown in a German clinical trial, which found that Olamkicept (gp130 trans-signaling inhibitor) could induce a clinical response in 44% and clinical remission in 19% of IBD patients; these patients showed a reduction in phosphorylated STAT3 [66]. Collectively, IL-6 is considered to be a pivotal tumor promoter in colitis-associated cancer and STAT3 is essential for the transduction of tumor-promoting signals from IL-6.

In addition to directly acting on intestinal epithelial cells (IECs) to induce STAT3 activation, IL-6 has also been shown to be an inducer of vascular endothelial growth factor receptor 2 (VEGFR2). Waldner et al. demonstrated that the complex of IL-6-dependent VEGFR2 and vascular endothelial growth factor (VEGF) could activate STAT3 to regulate angiogenesis, thus exerting a direct tumor-promoting effect [67] (Figure 1E). However, the activation of STAT3 can still be seen in IECs of IL-6^−/−^ mice, indicating that IL-6 is not the only activator of STAT3 [64]. Some studies found that TNF-α-activated sphingosine kinase 1 (SphK1) phosphorylated sphingosine to generate S1P, which could activate S1P receptor 1 (S1PR1), triggering the activation of the STAT3 [68,69] (Figure 1B). In turn, STAT3 is a direct transcription factor for the S1PR1 gene promoter; thus, SphK1/S1P/S1PR1 signaling could induce persistent STAT3 activation [70]. Based on these, multiple S1PR modulators are in clinical development (NCT04706793, NCT05061446, CTR20192294) for moderate to severe active UC, and have shown promise in phase II and III studies, with ozanimod is now approved for UC [71].

### 3.3. IL-23/Th17

IL-23/Th17 pathway appears to be an important route in UC-CRC transformation. In detail, IL-23, a heterodimeric cytokine produced by dendritic cells (DC) and macrophages, comprises p19 and p40 subunits [72]. Once p19 binds to the IL-23 receptor (IL-23R) via its N-terminal immunoglobulin domain, p19 helical domain occurs reconstruction that prompts p40 subunit to bind IL-12 receptor (IL-12R) β1 chain [73,74], which will then activate the JAK-STAT signaling cascade and finally promote expression of cytokines belonging to Th17 subtype, such as IL-17A, IL-21 and IL-22 [75] (Figure 1A).

Although many studies demonstrated that IL-17A is involved in tumorigenesis through activation of STAT3, some studies have demonstrated that IL-17A may not play a role depending on STAT3. Zepp et al. found that IL-17A regulated several target genes and showed the highest induction of placenta-expressed transcript 1 (Plet1) in the tumor. They analyzed Plet 1-deficient mice in the AOM/DSS model and found reduced tumor numbers and reduced expression of proliferation marker Ki67, suggesting IL-17A-induced Plet1 expression contributes to colon tumorigenesis and cell proliferation [76]. In addition, increased expression of IL-22, IL-22 receptor (IL-22R) and phosphorylated STAT3 was detected in intestinal mucosa in patients with UC or UC-CRC [77,78]. In cells enriched with IL-22, the C-terminal of IL-22R can recruit the coiled-coil domain of STAT3, and then in turn activates STAT3, inducing growth and metastasis-related genes (Bcl-xl, Cyclin D1 and VEGF) [77,79] (Figure 1C). Furthermore, evidence for the tumor-promoting role of IL-17A and IL-22 in UC-CRC was also verified in vivo. In the AOM/DSS-treated mice, a blockade of IL-17A by IL-17A antibody or a deficiency of IL-17A was linked to reduced tumor size and number [57,80,81]. Additionally, the number and size of the tumors were increased in IL-22 binding protein (a IL-22 receptor that prevents the binding of IL-22 to membrane-bound IL-22R) deficiency in mice [82]. Notably, evidence in colitis models also showed a protective effect of IL-22, which reflects the complex function of IL-22 related to intestinal inflammation. Overexpression of IL-22 triggered by thymopentin could restore the composition of probiotics such as *Lactobacillus* and *Akkermansia*, normalizing the composition of the gut microbiome, suggesting IL-22 may alleviate DSS-induced colitis [83]. Since it is not very clear why IL-22 functions as “a two-headed cytokine” and displays both proinflammatory and anti-inflammatory properties in maintaining gut homeostasis, further studies are required in the future to explain its mechanism.

IL-21, another cytokine secreted by Th17 cells, was overexpressed in patients with UC [84]. In colitis-associated cancer mice models, treatment of anti-IL-21 leads to lower numbers of tumors, and decreased colonic T cell infiltration, reduced IL-6 and IL-17A. Araki analyzed IL-21-isoform transgenic mice in an AOM/DSS model and found upregulation of activation-induced cytidine deaminase (AID) gene and protein. Overexpressed AID may promote carcinogenesis by causing genetic mutations and chromosomal abnormalities, and its tumor-promoting effect has also been reported in gastric cancer with *Helicobacter pylori* infection and liver cancer with hepatitis C viral infection [85]. In summary, these data suggest that IL-23/Th17 signaling provides a proinflammatory microenvironment that promotes tumor growth and progression.

### 3.4. COX-2/PGE2

When inflammation occurs in tissues, arachidonic acid-mediated by the enzyme COX-2 generates prostaglandin E2 (PGE2), which regulates multiple functions in immune cells by binding to the E-type prostanoid (EP) receptors. Of the four receptors (EP1, EP2, EP3, EP4 receptor), EP4 receptor is the most abundantly expressed subtype and is often upregulated during CRC [86]. In the 2,4,6-trinitrobenzene sulfonic acid (TNBS)-induced mice model, exacerbation of colitis caused by high doses of misoprostol (PGE2 analogs) or PGE1-OH (EP4 agonist) correlated with increased IL-23, a significant accumulation of Th17 cells, together with upregulation of other proinflammatory cytokines including IL-1β and IL-6. Thus, high levels of PGE2 may activate the IL-23/IL-17 axis by interacting with EP4 resulting in locally amplified and sustained inflammation [87]. Moreover, the crucial role of EP1 in CRC has also been demonstrated with AOM-treated mice, in which an EP1-selective antagonist (ONO-8711) reduced the numbers of intestinal polyps and diminished tumor development [88].

Additionally, the peroxisome proliferator-activated receptor δ (PPAR-δ) is also involved in the COX-2/PGE2-associated carcinogenesis. In APC^min^ mice, PGE2 treatment increased tumor burden by promoting cell survival while deficiency of PPAR-δ showed the opposite effect; this may rely on the activation of PI3K/AKT/PPAR-δ signaling induced by PEG2 [89] (Figure 1J). Of note, Wang et al. first provided in vivo evidence showing the reverse regulation of COX2 by PPAR-δ; this positive feedback loop can create a persistent proinflammatory microenvironment to increase the chance of tumorigenesis [90].

### 3.5. Wnt/β-Catenin

Aberrant Wnt/β-catenin signaling is central to sporadic CRC, in which approximately 80% of cases harboring APC gene mutations [91]. This mutation causes the accumulation of β-catenin in the nucleus, where it can upregulate the transcription of several target genes (e.g., AXIN2, SURVIVIN, ALDH, Cyclin D1) [92]. In contrast, the role of Wnt signaling in UC-CRC was not given much attention in the past, while recently it was found to be involved in tumor formation. For instance, YAP, the main component of Hippo signaling, was reported to form YAP/β-catenin/transcription factor 4 (TCF4) complex in the nucleus, which can upregulate intestinal stem cells (ISC) signature gene leucine-rich repeat-containing G-protein-coupled receptor 5 (Lgr5) and Cyclin D1 (Figure 1F). Functional evidence for this concept was offered by Deng et al., that the nuclear staining of YAP and β-catenin in the neoplastic area was enhanced in the mice model; conversely, phospho-mimetic YAP suppressed cancer cell proliferation and tumor development [93]. Additionally, nuclear translocation of β-catenin can be promoted through the indoleamine 2,3 dioxygenase-1 (IDO1)-kynurenine pathway (KP) mediated by PI3K/AKT signaling (Figure 1F). To determine the effect of IDO1 on tumor formation, Bishnupuri et al. studied mice with intestinal epithelial-specific deficiency of IDO1; they found that these mice developed fewer and smaller tumors compared with wild-type mice. In this AOM/DSS model, increased proliferation and decreased apoptosis were detected [94]. In addition to the role of tumor-promoting, Wnt/β-catenin could induce an inflammatory cascade by downregulating Claudin-7 (Cldn-7), which is responsible for TJs barrier integrity [95].

### 3.6. UC-CRC and Gut Microbiota

The involvement of gut microbiota in UC-CRC has gained much attention and growing evidence has indicated that it plays a role in the etiology of UC-CRC. Colitis-linked barrier defects enable bacteria and their degradation products to invade IECs. In this process, bacterial stimulation of TLR4 could activate signaling pathways involved in inflammatory responses through three main proteins: LPS-binding protein (LBP), CD14 and myeloid differentiation protein 2 (MD-2) [96]. Mechanically, LPS of Gram-negative bacilli could interact with LBP, transferring endotoxin to TLR4/MD-2 complex, which leads to the recruitment of adapter proteins MyD88 to the intracellular domain of TLR4, initiating the signal cascade and synthesis of IL-17C [97]. Ultimately, microbiota-driven IL-17C induces the expression of Bcl-2 and Bcl-xl in IECs to improve IECs survival and promote cancer development [98]. In line with this, TAK-242 (a small-molecule TLR4 inhibitor) strongly suppressed the development and progression of colonic tumors during DSS-induced colitis, with reduced numbers of infiltrating macrophages and colonic proinflammatory cytokine levels [99]. Moreover, for APC^Min/+^ MyD88^-/-^ mice, both mortality and tumor numbers were dramatically decreased [100]. Furthermore, since *Escherichia coli* (*E. coli*), *Fusobacterium nucleatum* (*F. nucleatum*) and *Enterotoxigenic bacteroides fragilis* (*ETBF*) have been shown to play an essential role in the development of CRC, we are here to focus on three bacteria and subsequently elucidate their correlation to UC-CRC.

Firstly, *E. coli* that harbors polyketide synthase (pks) was found more frequently in biopsies of CRC (66.7%) and IBD (40%) than in healthy individuals (20.8%) [101]. The pks codes for synthesizing colibactin (a genotoxin), which can induce DNA damage, cell cycle arrest, mutations and chromosomal instability in IECs, thus initiating cancer [102]. In both AOM/DSS and xenograft models, pks+ *E. coli* enhanced tumor growth via growth factor secretion, which is sustained by cellular senescence [103]. Based on this, restricting gut colonization with *E. coli* by oral administration of sodium tungstate decreased intestinal inflammation and reduced colibactin-driven tumorigenesis in AOM/DSS models [104]. More importantly, quantifying bacterial biomarkers may be a valuable tool to assist in solving intestinal disorders diagnostic challenges. Lopez-Siles et al. measured *Faecalibacterium prausnitzii* (*F. prausnitzii*) and *E. coli* abundance and calculated FP-E index, which was found to be an effective method to discriminate CRC and IBD [105]. Follow-up studies to establish if this index would be helpful to predict the risk of CRC associated with IBD or UC are needed.

Recently, *F. nucleatum* has been reported to be enriched in patients with UC and its abundance correlated with disease activity [106]. In preclinical experiments, Rubinstein et al. found that FadA (a virulence factor of *F. nucleatum*) could promote expression of inflammatory genes (e.g., NF-κB, IL-6, IL-8, IL-18) through E-cadherin/β-catenin signaling, thus generating a proinflammatory microenvironment that is conducive for CRC progression [107]. This proinflammatory effect has also been reported in several research articles [108,109,110]. Moreover, the importance of *F. nucleatum* in UC-CRC has attracted Yu et al.’s attention; they found that *F. nucleatum* could accelerate the progression of the tumor via epithelial–mesenchymal transition (EMT) both in vitro and in vivo. This promoting effect may lie in activating EGFR signaling pathways since EGFR inhibition downregulates EMT-associated molecules [111].

Finally, *ETBF* has been shown to induce ROS production and DNA damage through overexpressed spermine oxidase (SMO), thus propagating inflammation and tumorigenesis while inhibition of SMO reduces *ETBF*-induced colon tumorigenesis by 69% (*p* < 0.001) in the multiple intestinal neoplasia mouse model [112]. In the APC^min^ mice model, *ETBF* triggers IL-17-dependent NF-κB activation in colonic epithelial cells, resulting in distal colon tumorigenesis [113]. In addition, Cao et al. analyzed the HCT116 and SW480 cells infected by *ETBF*; they identified that downregulated miR-149-3p may facilitate Th 17 cell differentiation and target PHF5A to promote CRC cells proliferation, suggesting the measurement of miR-149-3p in plasma exosomes could be an effective approach to predict active UC and UC-CRC [114]. Considering the complexity of the gut microbiota, several studies have reported higher proportions of *γ-proteobacteria* and *Sphingomonas* genus, a lower proportion of *Ruminococcus* genus in colitis, whereas their effects on UC-CRC progression are still a mystery [115,116].

Considering the importance of dysbiosis associated with UC-CRC, multiple attempts are being undertaken to address the altered microbiota. Two studies found that *Lactobacillus gasseri* and *Bifidobacterium* reduced the risk of UC-CRC via regulation of inflammation, carcinogenesis and compositional change in gut microbiota [117,118]. Ten-week treatment of *Lactobacillus gasseri* to AOM/DSS-induced mouse model reduced both the incidence of colonic tumors and damage to the colonic mucosa effectively, with biomarkers associated with proinflammatory cytokines and inflammation-associated enzymes reduced, and anti-inflammatory cytokines increased [117]. Moreover, the administration of *Bifidobacterium* significantly attenuated tumor number in the mouse model and increased the relative abundance of multiple bacteria such as *Akkermansia*, *Desulfovibrionaceae*, *Romboutsia*, *Turicibacter*, *Verrucomicrobiaceae* [118]. In summary, microbiota plays an important but not entirely understood role in UC-CRC and is likely to be a promising strategy for diagnosis, prevention and therapy for UC-CRC.

## 4. Surveillance for CRC in UC

In order to facilitate clinical management effectively and to better predict which patients are more likely to develop carcinomas, it is critical that we put the surveillance of UC-associated dysplasia and cancer into effect [119]. Currently, we have given close attention to the timing of first surveillance, interval between colonoscopies, choice of techniques and biopsies for patients with chronic UC; multiple international societies have provided recommendations on these aspects [1,2,120,121].

### 4.1. Surveillance Strategies for CRC in UC

Most guidelines recommend the timing of the first screening colonoscopy based on the duration of time after symptom onset should be at 8–10 years [1,2,10] and specific intervals depending on risk stratification (high-, intermediate- and low-risk, determined by clinical characteristics of patients or disease) [2,122].

High-risk patients are advised to be monitored annually. An early cohort study assessed the importance of familial CRC in patients with IBD. The result showed that IBD patients with a first-degree relative (FDR) diagnosed with CRC before 50 years old had a high relative risk (RR 9.2, 95% CI 3.7–23) [123], which laid the foundation for later guidelines to stipulate that FDR diagnosed with CRC under the age of 50 was a high-risk factor among IBD patients [2,10,120]. Moreover, the increased risk of CRC in IBD-PSC patients has been proven. It is generally recommended that IBD patients with PSC should be monitored at the time of diagnosis, and then monitored annually [1,124,125]. Additionally, if dysplasia is diagnosed, patients with IBD tend to have a risk of developing CRC in the near future [35,36]. As such, surveillance is recommended yearly, if concerning dysplasia lesions (within 5 years) are detected, either HGD or LGD [2,10,37]. Extensive colitis with severe active inflammation is also classified as high risk. These data support recommendations from the main guidelines that the high-risk groups (e.g., extensive colitis with severe active inflammation, CRC in FDR aged <50 years, PSC, history of dysplasia within 5 years) deserve annual surveillance colonoscopy.

Intermediate-risk patients are advised to have colonoscopy surveillance every 2–3 years. IBD patients with an FDR diagnosed with CRC after 50 years old remain have a lower but consistently elevated RR (1.7, 95% CI 1.4–4.4) risk [123]. Nevertheless, regarding some other intermediate-risk factors that remain controversial, recommended surveillance intervals vary. In terms of inflammation, it is generally accepted that mildly active endoscopic/histologic inflammation was an intermediate-risk factor. Differently, moderate active endoscopic/histologic inflammation was classified as an intermediate-risk factor in ECCO guidelines (UC only). In contrast, it is classified as a high-risk factor in AGA consensus and British Society of Gastroenterology (BSG) grade, probably owing to the differing targeted people [2,10,37]. 

Furthermore, a colonoscopy every 5 years was required for patients with low-risk factors, which includes left-sided colitis and without active inflammation in BSG guideline [37], continuous disease remission since the last colonoscopy with mucosal healing on the current exam, plus either two consecutive exams without dysplasia or minimal historical colitis extent in AGA consensus [10], not high- or intermediate-risk factors in ECCO [2].

Epidemiological studies show poor compliance with endoscopic surveillance in patients with UC. According to the ECCO guidelines, a longitudinal, multicenter cohort study divided 1031 patients (732 UC, 259 CD and 40 indeterminate colitis) into low-, intermediate- and high-risk groups. Among them, 13%, 34% and 39% of patients did not perform or delayed follow-up procedures, respectively (*p* < 0.001). Groups at higher risk of CRC are associated with lower adherence [126]. The possible reasons mentioned may be transportation, finances and discomfort with the procedure or bowel preparation as barriers to endoscopic surveillance adherence [127]. Although from the perspective of cost-effectiveness, screening programs for all patients with IBD may be challenging. Even with authoritative guidelines, there is still room for improvement. A multicenter study showed that among the patients with IBD ongoing surveillance who had at least two consecutive colonoscopies demonstrating histologically quiescent disease and in the absence of high-risk features, the risk of aCRN with a median 6.1 years of follow-up was extremely low [128]. This suggests that an individualized approach is necessary for determining the timing of screening initiation and subsequent surveillance intervals. There is still a long way to find guidance to combine the above risk stratification with the number of risk factors for CRC and realize personalized management.

### 4.2. Comparison between Surveillance Techniques

Since the significance of screening and surveillance for patients with UC has been widely proven, several endoscopic techniques have been developed to identify dysplasia or cancer [1,2,12] (Table 1).

As is known to all, dysplasia in IBD is thought to be flat and difficult to detect. In the beginning, the recommended screening modality was white light endoscopy (WLE) with random four-quadrant biopsies every 10 cm for a total of 33 or more biopsies. Standard-definition WLE (SD-WLE) is an old technology, which was later confirmed inferior to high-definition WLE (HD-WLE) in SCENIC guidelines [129]. A retrospective analysis of 160 colonoscopies in the SD-WLE group and 209 colonoscopies in the HD-WLE group between 2008 and 2010 showed that HD-WLE significantly detected more dysplastic lesions than SD-WLE in patients with long-standing IBD [130]. For WLE, high-resolution equipment offers an opportunity to better identify and define the boundaries of tumor lesions because it has a wider visual field and a higher pixel density.

Chromoendoscopy (CE), another recommended technology, applies methylene blue or indigo carmine to the colon epithelium, enhancing areas of mucosal irregularity and delineating borders of suspected lesions. The SCENIC guideline suggested that CE is recommended rather than WLE when performing surveillance with SD or HD colonoscopy [129]. In terms of SD colonoscopy, a prospective trial enrolled 68 patients with IBD and concluded that CE was superior to SD-WLE in detecting dysplasia in IBD (OR 2.4, 95% CI 1.4–4.0) [131]. A recent meta-analysis of 10 studies was also published to assess the comparative efficacy of CE vs. SD-WLE, which confirmed that CE was more effective at identifying dysplasia than SD-WLE (RR 2.12, 95% CI 1.15–3.91) [132]. A prospective surveillance study randomized patients with long-standing IBD for HD colonoscopy and reported that HD-CE detection yield was superior to HD-WLE (17/152 vs. 7/153, *p* = 0.032) [133]. Although HD-CE is considered to be better than HD-WLE, some studies have shown that they are comparable. In a multicenter prospective randomized clinical trial (RCT), 210 subjects with long-standing UC were assigned to undergo HD-WLE (*n* = 102) and HD-CE (*n* = 108), and the dysplasia detection between the two groups was similar (3.9% in the HD-CE group vs. 5.6% in the HD-WL group, *p* = 0.749) [134]. This different finding might be caused by disparate inclusion criteria and the diversity of the population. Despite different views on whether HD-CE is better than HD-WLE, the advantages of CE in detecting lesions cannot be ignored.

With the development of endoscopic imaging technology, virtual chromoendoscopy (VCE) has also been popularized, which allows the use of CE without spraying dye agents, including narrow-band imaging (NBI), Fuji intelligent color enhancement (FICE) and i-Scan. A recent meta-analysis of 11 RCTs confirmed that VCE performed similarly to CE and HD-WLE concerning dysplasia detection on a per-patient basis [135]. In detail, NBI utilizes optical digital techniques to filter white light toward blue and green to enhance the visualization of vascular patterns and architecture of colonic mucosa without staining and it only requires one press on a particular button to reveal a suspicious lesion. A multicenter prospective randomized controlled trial was conducted in Belgium with a sample of 131 UC patients undergoing CE (*n* = 66) or NBI (*n* = 65). The result was that based on similar detection rates for the dysplasia between CE (21.2%) and NBI (21.5%) (OR 1.02, 95% CI 0.44–2.35, *p* = 0.964), NBI reduced an average of 7 min to procedural time (*p* < 0.001) [136], indicating that NBI may possibly be a convenient surveillance colonoscopy in patients with UC. I-Scan and FICE emphasize subtle changes in the mucosal surface through the reconstruction of a certain wavelength (computed spectral estimation technology). Iacucci et al. reported that the accuracy of CE, HD-WLE and i-Scan in predicting histological determination of neoplastic lesions was 81.4%, 86% and 78%, and the duration of the median withdrawal time in minutes was 16.2, 15.4 and 15.3. Moreover, dysplasia detection rates were similar among the three arms of the study during IBD surveillance colonoscopy (*p* = 0.84) [137]. It is the first study utilizing the new generation of i-Scan in surveillance IBD patients. However, the research on i-Scan is limited; multicenter prospective studies are required to verify the sufficiency for detection of dysplasia. The CONVINCE trial is an unblinded randomized delayed crossover trial of CE vs. FICE to detect dysplasia, enrolling 48 IBD patients. Dysplasia diagnostic accuracy of FICE and CE was 93.94% (95% CI 85.2%–98.32%) vs. 76.9% (95% CI 66.9%–98.2%), while examination time was 14 ± 4 vs. 20 ± 7 (95% CI 3.5–8, *p* < 0.001), which means dysplasia detection of FICE is at least as good as that of CE, and FICE has the advantage of being used for surveillance [138]. This trial served as a foundation for a multicenter trial to confirm the value of FICE for surveillance. From this research, it is not difficult to find that the efficacy of VCE and CE is similar. However, especially for patients who possessed high-risk factors requiring more frequent surveillance, they preferred VCE to DCE because the former has a shorter exploration time, less bloating or cramping and does not need extra cost for methylene blue/indigo carmine [138]. According to these data, the AGA clinical guideline has recommended that VCE can be a suitable alternative for dysplasia detection in persons with IBD [10].

Autofluorescence imaging (AFI) is a relatively novel technique that highlights neoplastic tissue without administrating exogenous fluorophores. Tissues exhibit fluorescence when exposed to ultraviolet (<400 nm) or shorter waveband visible light (primary blue). According to the CE vs. AFI for neoplasia detection in patients with longstanding UC (FIND-UC) randomized controlled trial, dysplasia was detected in 12% (13/105) patients by AFI and in 19% (20/105) patients by CE, with a relative detection rate of 0.65 (80% CI: 0.43–0.99). It seems AFI and CE are equally effective for detecting of dysplastic lesions in patients undergoing colonoscopic surveillance for UC, while the relative detection rate has not yet met the criteria (0.66) for noninferiority study, indicating AFI should not be an alternative to CE [139]. Another FIND-UC trial showed the value of AFI for excluding dysplasia lesions in long-standing UC, with 98.7% negative predictive value of AFI and 94.7% (95% CI 85.2%–98.32%) of CE [140]. Though the high negative predictive value of AFI, it should be noted that a large-scale, prospective study will still be needed to assess its place in the surveillance of UC patients.

The techniques mentioned above allow for the identification of mucosal lesions. Still, it is difficult to accurately diagnose intraepithelial neoplasia (IN) in UC accurately because of lacking cellular resolution and subsurface imaging. Confocal laser endomicroscopy (CLE) and endocytoscopy (EC) are promising tools that enable the acquisition of microstructural objects (e.g., capillaries, villiform structure, crypt, goblet, or epithelial cells, nucleus and cytoplasm), representing that endoscopy will shift from macro to micro, and from surface to deep [141]. As early as 2007, several studies have indicated that CLE could increase the diagnostic yield of INs in UC patients [142], which was later reported in 2008 [143]. In Kiesslich’s RCT, 153 UC patients randomly underwent either conventional colonoscopy or chromoscopy-guided CLE. According to this study, CLE increased neoplasia detection by 4.75 times, although 50% fewer biopsies were required. The sensitivity, specificity and accuracy of CLE reached 94.7%, 98.3% and 97.8%, respectively [142]. Consistent with the result of complete RCTs, a meta-analysis also revealed that CLE had a pooled sensitivity of 91% (95% CI 66%–98%), specificity of 97% (95% CI 94%–98%), which is a highly accurate technology for differentiating the neoplastic lesions from non-neoplastic lesions in patients with colonic IBD [144]. Although CLE can easily identify dysplastic regions of the mucosal layer and guide targeted endoscopic interventions, it has limited applicability. It cannot cover the whole gastrointestinal tract because it can only see a limited field of vision [145]. Therefore, before using CLE, it is necessary to use other macroscopic techniques such as CE to find suspicious areas and targeted biopsies. Additional obstacles to the universal use of CLE include high cost, long program time and the need for additional equipment. More researches are needed to verify the effectiveness of CLE in daily practice. EC is an emerging ultrahigh magnification endoscopic technique designed to provide excellence in vivo assessment of lesions found in the gastrointestinal tract. When used with intraprocedural stains, EC enables microscopic visualization of the gastrointestinal mucosal surface to obtain an ultramagnification pathological image simply by applying the scope to the target mucosa during an endoscopic examination [146]. Kudo’s team began exploring EC long years ago and established EC classification to differentiate between neoplastic and non-neoplastic lesions. Meanwhile, EC showed significantly higher diagnostic accuracy for predicting neoplastic and non-neoplastic lesions than conventional endoscopy (*p* = 0.015) [147]. Their study was only in the case of colorectal neoplasms in non-UC patients at this stage. They recently did a further step research on predicting UC-associated neoplasia (UCAN) under EC. Each lesion was diagnosed by two strategies: pit pattern (PIT) alone and a combination of EC-irregular nuclei to PIT (EC-IN-PIT). Results showed that EC-IN-PIT had improved the predictive values, especially for the specificity (84% vs. 58%, *p* < 0.001) and accuracy (88% vs. 67%, *p* < 0.01) compared with the PIT alone [148]. This is the first study to demonstrate the clinical impact of EC nuclear irregularities on predicting UCAN. Its value in cancer monitoring remains to be discussed.

In addition to the above techniques, full-spectrum endoscopy (FUSE) is a novel HD endoscopic technique that incorporates camera lenses to the colonoscopy tip’s right and left sides and the forward-viewing lens. This configuration allows endoscopists to observe behind folds and blind spots. In a prospective crossover study of 52 patients, Leong et al. found that FUSE detected more dysplastic lesions than conventional forward-viewing colonoscopy (FVC) in patients with IBD [149]. There is little research on FUSE, and further exploration into dysplasia surveillance is needed.

**Table 1:** A comparison of various surveillance modalities used for detecting colorectal dysplasia in patients with IBD.

Study	Year	IBD Type	Type of Endoscopy	Number of Patients	Patients with Dysplasia, *n* (%)	Number of Dysplasia	Number of CRC Patients	*p* Value	Withdrawal Time (Minutes)
Subramanian et al. [130]	2008–2010	IBD	SD-WLE HD-WLE	160 (UC101/CD59) 209 (UC147/CD62)	8 (5.00) 24 (11.48)	11 (HGD1) 32 (HGD2)	2 5	0.03	- -
Marion et al. [131]	2005–2011	IBD	SD-WLE CE	68 (UC55/CD13)	11 (16.18) 27 (39.71)	11 27	- -	0.001	- -
Yang et al. [134]	2013–2015	UC	HD-WLE HD-CE	108 102	6 (5.56) 4 (3.92)	- -	- -	0.749	17.6 16.5
Alexandersson et al. [133]	2011–2016	IBD	HD-WLE HD-CE	153 (UC90/CD62/Indeterminate colitis1) 152 (UC96/CD54/Indeterminate colitis2)	7 (4.58) 17 (11.18)	7 (LGD5/HGD2) 24 (LGD21/HGD3)	0 0	0.032	17 ± 8 24 ± 11
Bisschops et al. [136]	2008–2013	UC	CE NBI	66 65	14 (21.21) 14 (21.54)	30 (LGD30) 20 (LGD19/HGD1)	0 1	0.964	27.0 18.5
Iacucci et al. [137]	2014–2016	IBD	HD-WLE CE i-SCAN	90 (UC42/CD44/ Indeterminate colitis4) 90 (UC43/CD47/ Indeterminate colitis0) 90 (UC44/CD45/ Indeterminate colitis1)	23 (25.56) 22 (24.44) 14 (15.56)	22 (LGD22) 15 (LGD15) 11 (LGD11)	0 1 0	0.84	15.4 16.2 15.3
Vleugels et al. [139]	2013–2017	UC	CE AFI	105 105	20 (19.05) 13 (12.38)	37 (36LGD/1HGD) 14 (14LGD)	1 0	0.18	25.1 18.0
Kiesslich et al. [142]	-	UC	SD-WLE CLE	73 80	4 (5.48) 11 (13.75)	4 (3LGD/1HGD) 19 (12LGD/7HGD)	0 0	0.097	- -
Leong et al. [149]	2014–2015	IBD	FVC FUSE	27 (UC15/CD12) 25 (UC14/CD11)	2 6	7 19	0 0	0.018	12.0 15.8

IBD: inflammatory bowel disease, UC: ulcerative colitis, CD: Crohn’s disease, HD-WLE: high-definition white-light endoscopy, SD-WLE: standard-definition white-light endoscopy, CE: chromoendoscopy, NBI: narrow band imaging, AFI: autofluorescence imaging, CLE: confocal laser endomicroscopy, FVC: forward-viewing colonoscopy, LGD: low-grade dysplasia, HGD: high-grade dysplasia.

### 4.3. Comparison of Random Biopsies (Rb) vs. Targeted Biopsies (Tb)

There was no agreement on whether Rb or Tb was performed when doing endoscopic monitoring with the appearance of advanced imaging techniques. As we know, it is not always easy to endoscopically identify UC-associated CRC or dysplasia. Rb is used for surveillance colonoscopy to find these invisible lesions, in which random four-quadrant biopsies every 10 cm for a total of 33 or more biopsies are obtained. In Tb, specimens are obtained only when endoscopic findings indicate the possibility of neoplasia, leading to a smaller number of samples. CE with Tb was recommended in the 2017 ECCO consensus to detect dysplasia in IBD patients. Alternatively, Rb and Tb of any visible lesion should be performed if WLE is used [2]. In a multicenter RCT, Watanabe et al. observed that the dysplasia detection rate was similar between Tb and Rb (11.40% vs. 9.35%, *p* = 0.617). Nevertheless, the targeted group had less examination time than the random group among samples (26.6 vs. 41.7 min, *p* < 0.001), which means Tb appeared to be a more time-effective and cost-effective approach [150]. Consistent with the observation in the RCT, a study published by Gasia et al. in 2016 also suggested that Tb were preferred over Rb in HD colonoscopy, CE and VCE. Neoplastic lesions were detected in 8.2% of the procedures performed in the random biopsy group (95% CI 5.6–11.7) and 19.1% of procedures in the targeted biopsy group (95% CI 13.4–26.5, *p* < 0.001) [151]. However, the value of Rb cannot be ignored either. One study analyzing 71 patients with UC with coexisting PSC showed that neoplasia was identified in 22 colonoscopies by targeted or random biopsies, among which 10 (45.5%) neoplasia were detected only in random biopsies. This suggested that random biopsies should be considered in the presence of PSC in UC patients [152]. In addition to PSC, a large retrospective study involving 1000 IBD patients (495 UC, 505 Crohn’s colitis) reported that a familiar history of neoplasia, tubular-appearing colon and presence of PSC is associated with dysplasia detected by random biopsies. The yield of neoplasia by Rb only was 0.2% per biopsy (68/31865), but 12.8% per patient with neoplasia (12/94) [153]. Indeed, patients with high-risk characteristics may benefit from continuing the practice of Rb during surveillance examinations. Thus, Rb might be a more reassuring choice for patients with high-risk factors such as personal history of neoplasia, coexisting with PSC or colonic tubular changes. However, further data would be needed to confirm these findings.

## 5. Chemoprevention of CRC in UC

Several guidelines argue that optimal disease control with chemoprevention, which refers to the use of medical therapy, is imperative to minimizing an individual’s lifetime risk of developing CRC [1,2,37]. Potential main prevention agents in UC-CRC include 5-Aminosalicylic acid (5-ASA), immunomodulators, ursodeoxycholic acid (UDCA) and anti-TNF drugs (Table 2). Additionally, other medications have been evaluated, including nonsteroidal anti-inflammatory drugs, acetylsalicylic acid, statins, folic acid and calcium/vitamin D supplements. Still, there are not enough convincing trials to illustrate their role in chemoprevention of CRC in UC patients.

### 5.1. 5-ASA

As UC-CRC is believed to arise from long-standing chronic inflammation, patients who use anti-inflammatory medications might be relatively protected from developing CRC. 5-ASA negatively regulates the COX-2/PGE2 axis, inhibits EGFR, NF-κB and Wnt/β-catenin signaling [154]. It is used as the first-line therapy in patients with mild UC or moderate UC patients without poor prognostic factors; otherwise, biologics should be preferred [155]. The ECCO guideline published in 2017 for UC-CRC management recommended that the use of 5-ASA can be considered for lifelong chemoprevention [2], and a series of clinical trials confirmed this protective effect of 5-ASA in UC-CRC [156,157,158,159]. Similarly, the risk of CRN is also decreased by treatment with 5-ASA [160]. In a meta-analysis involving thirty-one independent observational studies, the analysis revealed a strong protective association between the use of 5-aminosalicylates and CRN in IBD patients (RR 0.57, 95% CI: 0.45–0.71) [161]. Although the impact of exposure to 5-ASA was favorable in patients with UC, a recent population-based cohort study in Hong Kong included 2103 IBD patients (1246 UC and 857 CD) demonstrated that the use of 5-ASA was not associated with a reduced risk of cancer development after adjustment for age, gender and smoking status (adjusted hazard ratio (aHR) 1.22, 95% CI 0.60–2.48, *p* = 0.593) [162]. One possible explanation may be related to medication adherence, as previous studies showed that only 40%–60% of patients had good compliance with it, and poor medication adherence may underestimate the effect of the drug [163,164]. Although there are different voices, the protective role of 5-ASA in UC-CRC is certain. However, there are still many unresolved questions such as the role of a combination therapy with 5-ASA in cancer prevention, how to improve adherence issues with 5-ASA therapy and whether UC patients should continue regular 5-ASA after achieving endoscopic remission solely for chemoprotective effect. Future prospective evaluation trials should focus continuously on these questions.

### 5.2. Thiopurines

The research on thiopurine usage in preventing the development of dysplasia or CRC in patients with UC is limited and controversial. As we all know, it has a chemopreventive effect by reducing colonic inflammation and promoting mucosal healing in the gastrointestinal tract. The early published studies failed to prove the chemopreventive effect [165,166], while most recent studies showed the opposite. In a retrospective study that included 831 UC patients, a decreased risk of colorectal neoplasms was observed in patients with long-term UC after treatment with thiopurines compared to patients who were never treated with thiopurines (OR 0.21, 95% CI 0.06–0.74, *p* = 0.015) [167]. A meta-analysis of 76,999 patients evaluating the correlation between thiopurine therapy and CRN risk showed significant protective effects in UC patients (OR 0.67; 95% CI 0.45–0.98) [168]. Nonetheless, while it has a protective effect, the tumor risk cannot be ignored simultaneously. There is evidence that it can increase the risk of malignancies like nonmelanoma skin cancer, lymphomas and urinary tract tumor through its immunosuppression, DNA-structure destruction, interference with DNA replication and repair and mutagenicity [169,170,171]. Therefore, reservations remain about their use in reducing the CRC risk. ECCO guidelines stated that there was insufficient evidence to recommend either for or against chemoprevention with thiopurines [1,2]. 

### 5.3. Ursodeoxycholic Acid

As mentioned above, high levels of bile acids in colon patients with both IBD and PSC may have carcinogenic effects, ultimately leading to dysplasia or CRC [11,24,172]. Previous RCTs have focused on the curative effect of UDCA in this population and proved that UDCA significantly decreases the risk of developing CRC and colorectal dysplasia in patients with UC and PSC [173,174]. UDCA is a steroid bile acid that increases bile flow and changes the hydrophobic index of the bile acid pool, which is also reported to have immunosuppressive and antitumorigenic effects [175]. A systematic review and meta-analysis showed a significant chemopreventive effect on the risk of aCRN (colorectal cancer and/or high-grade dysplasia) (OR 0.35, 95% CI 0.17–0.73). In a subgroup analysis, low-dose UDCA use (8–15 mg/kg/d) was also associated with a significant risk reduction in CRN (OR 0.19; 95% CI, 0.08–0.49) [172]. Current guidelines recommend against using UDCA for the sole purpose of preventing CRC [2,37], and further studies of the chemoprotective effects of UDCA in the larger population with UC but not PSC and the effect of low and high dose UDCA are needed.

### 5.4. Biologics

In addition to traditional therapies, biological therapies with anti-TNF agents (such as infliximab, adalimumab and golimumab) are used for patients with moderate-to-severe UC who have an inadequate response to conventional therapy, which raise the therapeutic efficacy simultaneously in modifying the disease course and preventing cancer [176]. It has been shown that anti-TNF-α agents can reduce UC-CRC occurrence by inhibiting chronic inflammation-induced NF-kB activation in IECs [177]. Based on the view of effective risk reduction, we spotted the data from a multicenter study from America suggesting that exposure to anti-TNFα agents in UC patients is associated with a reduction in CRC risk after adjustment for possible confounding factors (OR 0.78, 95% CI 0.73–0.83, *p* < 0.0001) [14], which was confirmed in other studies additionally [178,179]. Although anti-TNF has acceptable safety profiles, there are concerns about the risk of adverse events, including infections, malignancy, metabolic and immunological diseases [180]. Moreover, other drugs such as Ustekinumab targeted IL-12/IL23, Vedolizumab targeted integrin α4β7 and Tofacitinib targeted JAK are approved to treat UC [181,182,183]. Since the anti-inflammatory effects of these agents have been demonstrated, it is reasonable to speculate that they could also be applied to UC-CRC.

**Table 2:** Studies assessing potential main prevention agents in IBD patients.

Drugs	Study Design	Area	Study Population	Primary Outcome	OR/RR/HR	95% CI	*p* Value	Effect	Ref.
**5-ASA**	Case–control study	French	IBD	CRC	0.59	0.37–0.94	0.026	Protective	[156]
	Case–control study	Finland	IBD	CRC	0.17	0.03–1.01	0.051	Protective	[157]
	Case–control study	UK	UC	CRC	0.60	0.38–0.96	-	Protective	[158]
	Cohort study	China	IBD	CRC	1.22	0.60–2.48	0.593	No effect	[162]
	Case–control study	-	IBD	CRN	0.27	0.08–0.88	-	Protective	[160]
	Case–control study	Asian	UC	CRC	0.25	0.06–1.02	0.037	Protective	[159]
	Meta-analysis	-	IBD	CRN	0.57	0.45–0.71	-	Protective	[161]
**Thiopurine**	Cohort study	Spanish	UC	CRN	0.21	0.06–0.74	0.015	Protective	[167]
	Case–control study	French	IBD	CRC	0.76	0.43–1.34	0.347	No effect	[156]
	Case–control study	Netherlands	IBD	CRC	0.30	0.16–0.56	<0.001	Protective	[178]
	Case–control study	-	IBD	CRN	0.18	0.05–0.70	-	Protective	[160]
	Case–control study	USA	UC	CRN	1.06	0.59–1.93		No effect	[166]
	Case–control study	Finland	IBD	CRC	0.09	0.02–0.33	0.0003	Protective	[157]
	Meta-analysis	-	UC	CRN	0.67	0.45–0.98	0.006	Protective	[168]
	Meta-analysis	-	IBD	CRN	0.71	0.54–0.94	0.017	Protective	[184]
**Ursodeoxycholic acid**	Cohort study	USA	UC	CRC	0.59	0.26–1.36	-	No effect	[185]
	Case–control study	USA	UC	CRC	0.26	0.06–0.92	0.034	Protective	[173]
	Meta-analysis	-	IBD	aCRN	0.35	0.17–0.73	-	Protective	[172]
**Anti-TNFα agents**	Cohort study	USA	UC	CRC	0.78	0.73–0.83	<0.0001	Protective	[14]
	Case–control study	Netherlands	IBD	CRC	0.09	0.01–0.68	<0.02	Protective	[178]
	Cohort study	French	UC	CRC	0.41	0.20–0.86	-	Protective	[179]

CRC: colorectal cancer, CRN: colorectal neoplasia, aCRN: advanced colorectal neoplasia.

## 6. Conclusions

Taken together, long-standing chronic inflammation in the intestinal mucosa increases the risk of CRC due to genetic and molecular changes. Identifying high-risk groups and surveillance endoscopy programs have been established to prevent this outcome. Although our understanding of the molecular mechanisms, management and prevention of UC-CRC has achieved dramatic advancement, several areas need to be further investigated: (1) more effective time intervals and tailored monitoring should be based on individual risk profile; (2) development of noninvasive biomarkers to avoid unnecessary colonoscopy; (3) a better understanding of the mechanisms may help reduce tumorigenesis through acting on novel targets; (4) prospective head-to-head trials regarding chemoprevention agents are eagerly awaited to provide more robust evidence. Thus, further studies on the involvement of intestinal inflammation, surveillance and prevention strategies in UC-CRC are required in the future.
